# Supplementary material for: An open-source framework for large-scale, flexible evaluation of biomedical text mining systems
Source: J Biomed Discov Collab. 2008 Jan 29;3:1. doi: 10.1186/1747-5333-3-1 (PMC2276192; doi:10.1186/1747-5333-3-1)
Supplement: Additional file 1 — Evaluation platform source code. The additional file contains the software evaluation framework discussed in this paper. The most current release can be downloaded from SourceForge.net [1]. See the accompanying README file for instructions on installation and use. Also included in the distribution is a collection of UIMA wrappers for some commonly used BioNLP tools and annotated corpora. [file 1747-5333-3-1-S1.zip › bionlp_uima_repository-1.0.1/docs/api/src-html/edu/uchsc/ccp/util/nlp/annotation/comparison/SpanComparator.html]

```
001    package edu.uchsc.ccp.util.nlp.annotation.comparison;
002    
003    import java.util.Collections;
004    import java.util.Comparator;
005    import java.util.List;
006    
007    import edu.uchsc.ccp.util.nlp.annotation.InvalidSpanException;
008    import edu.uchsc.ccp.util.nlp.annotation.Span;
009    
010    public abstract class SpanComparator implements Comparator<Span> {
011    
012        /**
013         * This method will be overridden by subclasses of SpanComparator
014         */
015        public abstract int compare(Span span1, Span span2);
016    
017        /**
018         * 
019         * This method will be overridden by subclasses of SpanComparator
020         * 
021         * @param spanList1
022         * @param spanList2
023         * @return
024         */
025        public abstract int compare(List<Span> spanList1, List<Span> spanList2);
026    
027        /**
028         * Returns 0 if the spans are equal, -1 if span1 starts before span2, 1 if span1 starts after span2. <br>
029         * If the spans start at the same index, then -1 if span1 ends before span2 and 1 if span1 ends after span2
030         */
031        public int matches(Span span1, Span span2) {
032            if ((span1.getSpanStart() == span2.getSpanStart()) & (span1.getSpanEnd() == span2.getSpanEnd())) {
033                /* if the spans are equal, then return 0 */
034                return 0;
035            } else if (span1.getSpanStart() == span2.getSpanStart()) {
036                /* if the spans have equal span start indexes, then return -1 if span1 ends before span2, 1 otherwise */
037                if (span1.endsBefore(span2)) {
038                    return -1;
039                } else {
040                    return 1;
041                }
042            } else {
043                /* the spans are not equal, and they don't have the same span start indexes, so return -1 if span1 starts before span2, 1 otherwise */
044                if (span1.startsBefore(span2)) {
045                    return -1;
046                } else {
047                    return 1;
048                }
049            }
050        }
051    
052        /**
053         * Compares a list of spans. If the lists have identical contents, return 0. If spanList1 starts before spanList2, return -1, otherwise return 1.<br>
054         * Note: an empty list will also return 0.
055         */
056        public int matches(List<Span> spanList1, List<Span> spanList2) {
057            SpanComparator sc = new StrictSpanComparator();
058            /* sort the lists just to be certain */
059            Collections.sort(spanList1, sc);
060            Collections.sort(spanList2, sc);
061    
062            // if (spanList1.equals(spanList2)) {
063            // /* if the lists are identical, return 0 */
064            // return 0;
065            // } else
066            if (spanList1.size() == 0 & spanList2.size() > 0) {
067                /* if list1 is empty, and list2 is non-empty, then return 1, as the empty list "comes after" the non-empty list */
068                return 1;
069            } else if (spanList1.size() > 0 & spanList2.size() == 0) {
070                /* if list1 is non-empty and list2 is empty, then return -1, as the non-empty list "comes before" the empty list */
071                return -1;
072            } else if (spanList1.size() == 0 & spanList2.size() == 0) {
073                /* if both lists are empty, consider them equal. */
074                return 0;
075            } else {
076                /* there are members in both span lists, so pick the first and last member, since it is sorted, and use them to determine what to return. */
077                Collections.sort(spanList1, sc);
078                Collections.sort(spanList2, sc);
079                Span firstSpanFromList1 = spanList1.get(0);
080                Span firstSpanFromList2 = spanList2.get(0);
081                Span lastSpanFromList1 = spanList1.get(spanList1.size() - 1);
082                Span lastSpanFromList2 = spanList2.get(spanList2.size() - 1);
083    
084                try {
085                    Span compositeSpan1 = new Span(firstSpanFromList1.getSpanStart(), lastSpanFromList1.getSpanEnd());
086                    Span compositeSpan2 = new Span(firstSpanFromList2.getSpanStart(), lastSpanFromList2.getSpanEnd());
087    
088                    return sc.compare(compositeSpan1, compositeSpan2);
089    
090                } catch (InvalidSpanException e) {
091                    e.printStackTrace();
092                    return -1;
093                }
094            }
095        }
096    
097        /**
098         * Returns 0 if span1 and span2 overlap. Otherwise, it returns -1 if span1 starts before span2, and 1 if span1 starts after span2
099         * 
100         * @param span1
101         * @param span2
102         * @return
103         */
104        public int overlaps(Span span1, Span span2) {
105            int s0start = span1.getSpanStart();
106            int s0end = span1.getSpanEnd();
107            int s1start = span2.getSpanStart();
108            int s1end = span2.getSpanEnd();
109            if ((s0start >= s1start & s0start < s1end) | (s0end > s1start & s0end <= s1end) | (s0start <= s1start & s0end > s1start) | (s0end >= s1end & s0start < s1end)) {
110                return 0;
111            } else {
112                return matches(span1, span2);
113            }
114        }
115    
116        /**
117         * Returns 0 if the span lists overlap. This method requires piecemeal overlap. That is, a list containing [1,5] [15,20] overlaps with a list
118         * containing [3,6] but does not overlap with the list: [6,11] [22,25]. Otherwise, it returns -1 if span1 starts before span2, and 1 if span1
119         * starts after span2
120         * 
121         * @param span1
122         * @param span2
123         * @return
124         */
125        public int overlaps(List<Span> spanList1, List<Span> spanList2) {
126            SpanComparator sc = new SloppySpanComparator();
127            if (spanList1.size() == 0 & spanList2.size() > 0) {
128                /* if list1 is empty, and list2 is non-empty, then return 1, as the empty list "comes after" the non-empty list */
129                return 1;
130            } else if (spanList1.size() > 0 & spanList2.size() == 0) {
131                /* if list1 is non-empty and list2 is empty, then return -1, as the non-empty list "comes before" the empty list */
132                return -1;
133            } else if (spanList1.size() == 0 & spanList2.size() == 0) {
134                /* if both lists are empty, consider them overlapping - though this is weird. */
135                return 0;
136            } else {
137                boolean spanOverlapDetected = false;
138                for (Span span1 : spanList1) {
139                    for (Span span2 : spanList2) {
140                        if (sc.compare(span1, span2) == 0) {
141                            spanOverlapDetected = true;
142                            break;
143                        }
144                    }
145                    if (spanOverlapDetected) {
146                        break;
147                    }
148                }
149                if (spanOverlapDetected) {
150                    return 0;
151                } else {
152                    return matches(spanList1, spanList2);
153                }
154            }
155        }
156    
157        /**
158         * Returns 0 if span1 and span2 share the same start index. Otherwise, it returns -1 if span1 starts before span2, and 1 if span1 starts after
159         * span2
160         * 
161         * @param span1
162         * @param span2
163         * @return
164         */
165        public int sharesSpanStart(Span span1, Span span2) {
166            if (span1.getSpanStart() == span2.getSpanStart()) {
167                return 0;
168            } else {
169                return matches(span1, span2);
170            }
171        }
172    
173        /**
174         * Returns 0 if span1 and span2 share the same start index. Otherwise, it returns -1 if span1 starts before span2, and 1 if span1 starts after
175         * span2
176         * 
177         * @param span1
178         * @param span2
179         * @return
180         */
181        public int sharesSpanStart(List<Span> spanList1, List<Span> spanList2) {
182            SpanComparator sc = new SharedStartSpanComparator();
183            if (spanList1.size() == 0 & spanList2.size() > 0) {
184                /* if list1 is empty, and list2 is non-empty, then return 1, as the empty list "comes after" the non-empty list */
185                return 1;
186            } else if (spanList1.size() > 0 & spanList2.size() == 0) {
187                /* if list1 is non-empty and list2 is empty, then return -1, as the non-empty list "comes before" the empty list */
188                return -1;
189            } else if (spanList1.size() == 0 & spanList2.size() == 0) {
190                /* if both lists are empty, consider them overlapping - though this is weird. */
191                return 0;
192            } else {
193                /* there are members in both span lists, so pick the first and last member, since it is sorted, and use them to determine what to return. */
194                Collections.sort(spanList1, sc);
195                Collections.sort(spanList2, sc);
196                Span firstSpanFromList1 = spanList1.get(0);
197                Span firstSpanFromList2 = spanList2.get(0);
198                Span lastSpanFromList1 = spanList1.get(spanList1.size() - 1);
199                Span lastSpanFromList2 = spanList2.get(spanList2.size() - 1);
200    
201                try {
202                    Span compositeSpan1 = new Span(firstSpanFromList1.getSpanStart(), lastSpanFromList1.getSpanEnd());
203                    Span compositeSpan2 = new Span(firstSpanFromList2.getSpanStart(), lastSpanFromList2.getSpanEnd());
204    
205                    return sc.compare(compositeSpan1, compositeSpan2);
206                } catch (InvalidSpanException e) {
207                    e.printStackTrace();
208                    return -1;
209                }
210            }
211        }
212    
213        /**
214         * Returns 0 if span1 and span2 share the same end index. Otherwise, it returns -1 if span1 starts before span2, and 1 if span1 starts after span2
215         * 
216         * @param span1
217         * @param span2
218         * @return
219         */
220        public int sharesSpanEnd(Span span1, Span span2) {
221            if (span1.getSpanEnd() == span2.getSpanEnd()) {
222                return 0;
223            } else {
224                return matches(span1, span2);
225            }
226        }
227    
228        /**
229         * Returns 0 if span1 and span2 share the same end index. Otherwise, it returns -1 if span1 starts before span2, and 1 if span1 starts after span2
230         * 
231         * @param span1
232         * @param span2
233         * @return
234         */
235        public int sharesSpanEnd(List<Span> spanList1, List<Span> spanList2) {
236            SpanComparator sc = new SharedEndSpanComparator();
237            if (spanList1.size() == 0 & spanList2.size() > 0) {
238                /* if list1 is empty, and list2 is non-empty, then return 1, as the empty list "comes after" the non-empty list */
239                return 1;
240            } else if (spanList1.size() > 0 & spanList2.size() == 0) {
241                /* if list1 is non-empty and list2 is empty, then return -1, as the non-empty list "comes before" the empty list */
242                return -1;
243            } else if (spanList1.size() == 0 & spanList2.size() == 0) {
244                /* if both lists are empty, consider them overlapping - though this is weird. */
245                return 0;
246            } else {
247                /* there are members in both span lists, so pick the first and last member, since it is sorted, and use them to determine what to return. */
248                Collections.sort(spanList1, sc);
249                Collections.sort(spanList2, sc);
250                Span firstSpanFromList1 = spanList1.get(0);
251                Span firstSpanFromList2 = spanList2.get(0);
252                Span lastSpanFromList1 = spanList1.get(spanList1.size() - 1);
253                Span lastSpanFromList2 = spanList2.get(spanList2.size() - 1);
254    
255                try {
256                    Span compositeSpan1 = new Span(firstSpanFromList1.getSpanStart(), lastSpanFromList1.getSpanEnd());
257                    Span compositeSpan2 = new Span(firstSpanFromList2.getSpanStart(), lastSpanFromList2.getSpanEnd());
258    
259                    return sc.compare(compositeSpan1, compositeSpan2);
260                } catch (InvalidSpanException e) {
261                    e.printStackTrace();
262                    return -1;
263                }
264            }
265        }
266    
267        /**
268         * Returns 0 if span1 and span2 share the same start or end index (or both). Otherwise, return -1 if span1 starts before span2, and 1 if span1
269         * starts after span2
270         * 
271         * @param span1
272         * @param span2
273         * @return
274         */
275        public int sharesSpanStartOrEnd(Span span1, Span span2) {
276            if ((sharesSpanStart(span1, span2) == 0) | (sharesSpanEnd(span1, span2) == 0)) {
277                return 0;
278            } else {
279                return matches(span1, span2);
280            }
281        }
282    
283        /**
284         * Returns 0 if span1 and span2 share the same start or end index (or both). Otherwise, return -1 if span1 starts before span2, and 1 if span1
285         * starts after span2
286         * 
287         * @param span1
288         * @param span2
289         * @return
290         */
291        public int sharesSpanStartOrEnd(List<Span> spanList1, List<Span> spanList2) {
292            SpanComparator sc = new SharedStartOrEndSpanComparator();
293            if (spanList1.size() == 0 & spanList2.size() > 0) {
294                /* if list1 is empty, and list2 is non-empty, then return 1, as the empty list "comes after" the non-empty list */
295                return 1;
296            } else if (spanList1.size() > 0 & spanList2.size() == 0) {
297                /* if list1 is non-empty and list2 is empty, then return -1, as the non-empty list "comes before" the empty list */
298                return -1;
299            } else if (spanList1.size() == 0 & spanList2.size() == 0) {
300                /* if both lists are empty, consider them overlapping - though this is weird. */
301                return 0;
302            } else {
303                /* there are members in both span lists, so pick the first and last member, since it is sorted, and use them to determine what to return. */
304                Collections.sort(spanList1, sc);
305                Collections.sort(spanList2, sc);
306                Span firstSpanFromList1 = spanList1.get(0);
307                Span firstSpanFromList2 = spanList2.get(0);
308                Span lastSpanFromList1 = spanList1.get(spanList1.size() - 1);
309                Span lastSpanFromList2 = spanList2.get(spanList2.size() - 1);
310    
311                try {
312                    Span compositeSpan1 = new Span(firstSpanFromList1.getSpanStart(), lastSpanFromList1.getSpanEnd());
313                    Span compositeSpan2 = new Span(firstSpanFromList2.getSpanStart(), lastSpanFromList2.getSpanEnd());
314    
315                    return sc.compare(compositeSpan1, compositeSpan2);
316                } catch (InvalidSpanException e) {
317                    e.printStackTrace();
318                    return -1;
319                }
320            }
321        }
322    
323    }
```
